# Contradictory effects of leaf rolls in a leaf-mining weevil

**DOI:** 10.1038/s41598-020-69002-1

**Published:** 2020-07-22

**Authors:** Chisato Kobayashi, Kazunori Matsuo, Masakado Kawata

**Affiliations:** 1 Graduate School of Life Sciences, Tohoku University, 3−6 Aoba, Aramaki, Aoba, Sendai, Miyagi 980-8578 Japan; 2 Biosystematics Laboratory, Faculty of Social and Cultural Studies, Kyushu University, 744 Motooka, Nishi, Fukuoka, Fukuoka 819‑0395 Japan

**Keywords:** Ecology, Evolution

## Abstract

Leaf rolls by herbivorous insects evolved in various lepidopteran groups, aphids, and some attelabid weevil species. Leaf rolls are known to have a positive effect on the survival of immature insects, protecting them from natural enemies such as birds, ants, predatory wasps, and parasitoids as well as environmental stress. On the other hand, leaf rolls are considered to have a negative effect on immature survival, attracting natural enemies because of their noticeability and subsequent learning or specialization. In this study, we directly tested the effects of leaf rolls using an attelabid species by comparing the fate of immature insects between artificial leaf rolls and unrolled leaves. The results showed the following positive effects of leaf rolls: avoidance of parasitism by eulophid wasps and avoidance of egg predation by unknown predators. On the other hand, a negative effect of leaf rolls was also detected, specifically and increase in mortality via leaf roll herbivory. This study indicated that leaf shelters are not only protective refuges but are also sometimes risky hiding places, although total survival rates increased in leaf shelters.

## Introduction

Herbivorous insects are one of the most diverse organisms in terrestrial ecosystems, and have evolved various types of feeding niches such as leaf chewers, leaf gallers, leaf miners, and stem borers^1–4^. Leaf roller is one of various feeding niches of herbivorous insects and many species in different taxa, for examples, tortricid moths, pyralid moths, gelechiid moths, aphids, and attelabid weevils, all show leaf rolling traits and grow in leaf rolls during their immature period or the adult period.

Leaf shelters, including leaf rolls, folds and ties, are known to have positive effects on immature survival by providing protection from predators such as birds, ants, spiders and wasps, as well as from environmental stress^1,5–15^. Additionally, it is indicated that leaf rolls have a protective effect against parasitic wasps^16^. In attelabid weevils, leaf rolling behavior by female insects to provide shelter and food for immatures evolved in the tribes Deporaini and Byctiscini (Rhynchitinae), and subfamily Attelabinae^17^. However, the effects of leaf shelters are only indirectly shown by interspecific comparison, experimental exposure to predators, or using dummy larvae, and few studies have shown the direct impact of leaf shelters under natural conditions. In contrast, leaf shelters may negatively affect immature insect survival, attracting natural enemies because of their noticeability^18,19^.

In Byctiscini and Attelabinae, leaf rolling behavior evolved once at the common ancestor. On the other hand, species of Deporaini are basically leaf miners of leaves cut by female insects, and leaf rolling behavior evolved several times independently, but most species remain non-leaf-rolling species. Thus, in Deporaini, leaf rollers and non-leaf rollers coexist within closely related species, suggesting that the leaf rolling behavior is not always preferred by natural selection, with selective pressure affected by subtle changes in conditions at the particular time and place. For example, females of *Deporaus betulae* construct a funnel-shaped open leaf roll using leaves of *Betula platyphylla* (Betulaceae), but females of the closely related species, *D. affectatus*, do not construct leaf rolls only cutting leaves of Betulaceae^17^. Similarly, females of *D. unicolor* construct cigar-like closed leaf rolls using leaves of some species of Fagaceae and Betulaceae, but females of *D. insularis*, do not construct leaf rolls, only cutting leaves of some evergreen Fagaceae^17^. Leaf-rolling attelabids suffer lower parasitism rates than non-leaf-rolling attelabids, and leaf-roll type and parasitoid communities are well associated; species with similar leaf roll types also show similar parasitoid communities even if they are not so closely related^16,17^. Thus, in Attelabidae, it appears that the evolution of leaf rolling behavior and leaf roll shape were greatly influenced by parasitoids.

**Figure 1:**
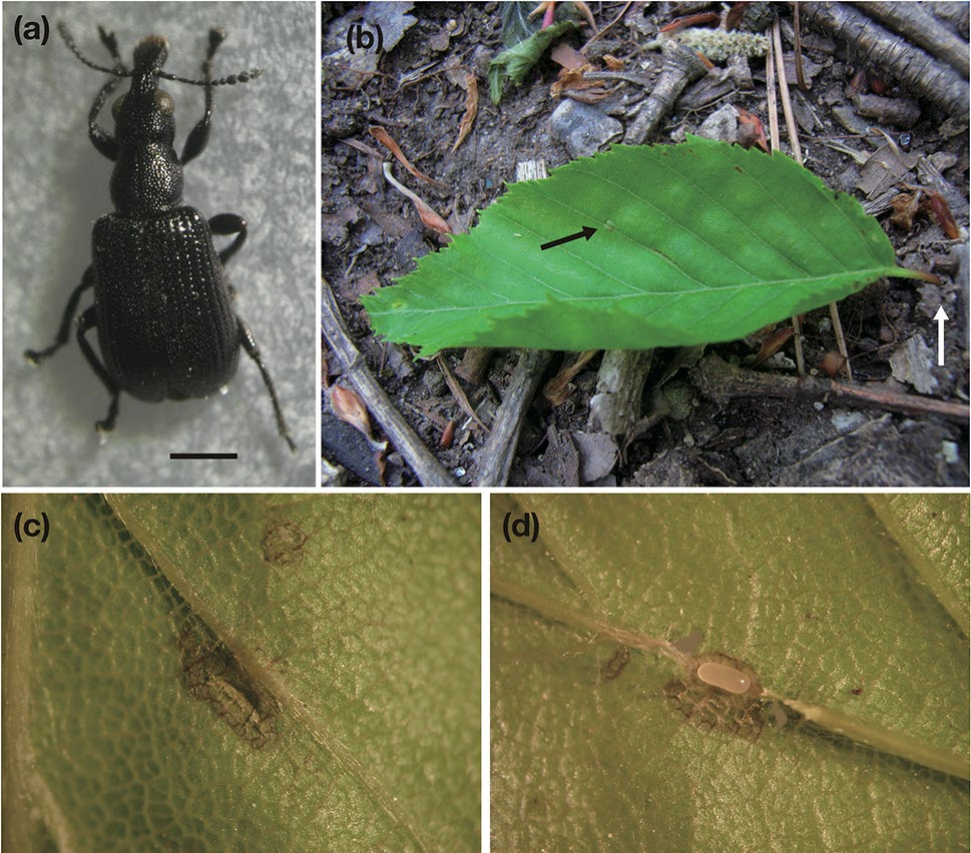
(**a**) Adult *Apoderites commodus* (Attelabidae, Deporaini), scale bar: 1 mm. (**b**) An oviposited and cut off leaf by a female *A*. *commodus* using *Carpinus laxiflora*. The black arrow shows an oviposition hole with an egg inside the leaf blade tissue. The white arrow shows a cut-off point of the petiole by a female *A*. *commodus*. (**c**) An oviposition slit made by a female *A*. *commodus*. An egg is embedded in the leaf blade tissue under the midrib. (**d**) An opened oviposition slit with an egg of *A*. *commodus*.

In this study, we evaluated the direct effect of leaf rolls using an attelabid weevil, *Apoderites commodus* (Deporaini, Attelabidae, Fig. 1), by comparing the immature fate between insects in artificial leaf rolls and those in unrolled control leaves in the field.

## Results

**Figure 2:**
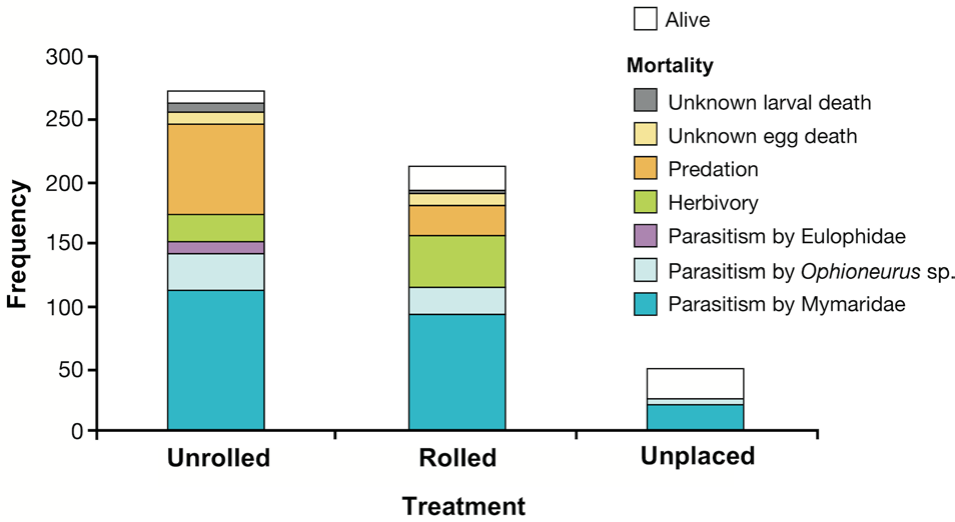
Frequencies of alive and dead eggs or larvae in unrolled, rolled and unplaced leaves. Mortalities were classified into seven categories: unknown larval death, unknown egg death, predation, herbivory, parasitism by Eulophidae, parasitism by *Ophioneurus* sp., and parasitism by Mymaridae.

Of the 50 eggs in the collected leaves (unplaced, Fig. 2), 22 (44.0%) died of parasitism by Mymarid wasps and 4 (8.0%) died of parasitism by *Ophioneurus* sp. Similarly, in the eggs of the recollected rolled and non-rolled leaves (control), 114 (41.8%) and 93 (43.9%) died of parasitism by Mymarid wasps, and 28 (10.3%) and 22 (10.4%) died of parasitism by *Ophioneurus* sp., respectively (Fig. 2). Both mymarid wasps and *Ophioneurus* sp. are solitary endoparasitoids of eggs. Parasitism rates of Mymaridae and *Ophioneurus* sp. were quite similar among the collected (unplaced) and recollected rolled and unrolled leaves with no significant difference (Fisher’s exact test, χ^2^ = 0.519, *df* = 4, *P* = 0.9716). This indicates that at the start of the experiment, approximately 40% of eggs had been parasitized by Mymarid wasps and approximately 10% had been parasitized by *Ophioneurus* sp. Thus, parasitism by Mymaridae and *Ophioneurus* sp. can be regarded as mortality before the experiment, so these mortalities were excluded from the following analysis comparing experimentally rolled leaves and unrolled leaves. Some emerged mymarid wasps were identified as *Anaphes* sp., but others could not be identified because of specimen damage.

**Figure 3:**
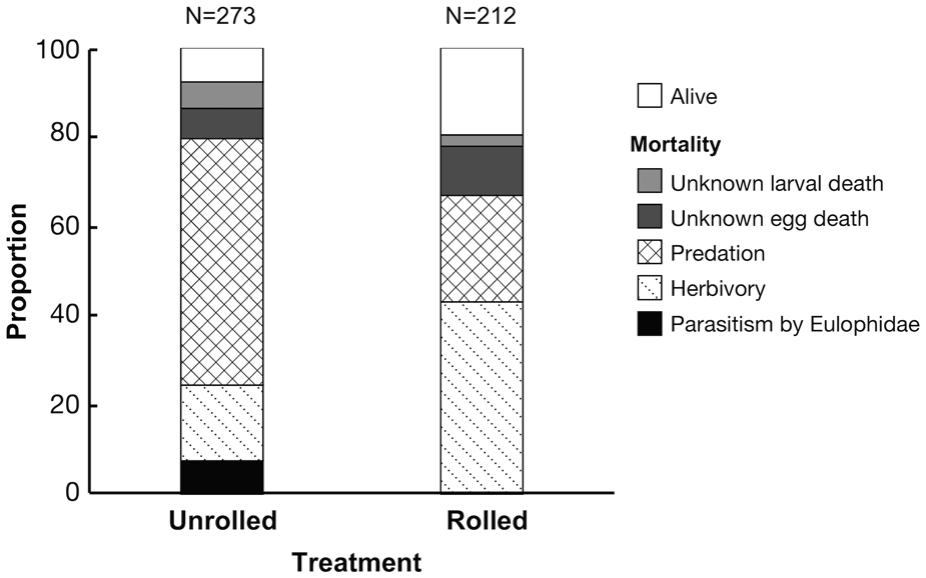
The proportion of living and dead immature weevils, where the cause of death was classified as unknown larval death, unknown egg death, predation, herbivory and parasitism by Eulophidae, in unrolled and rolled treatments.

Overall, the survival rate of immatures in experimentally rolled leaves was significantly higher (19.6%) than in non-rolled leaves (7.6%; bootstrap possibility, *P* < 0.001; Fig. 3). In experimentally rolled leaves, eggs or larvae were free from mortality by parasitism by Eulophidae, which are solitary endoparasitoids of eggs or larvae. On the other hand, in unrolled leaves, 7 eggs and 3 larvae (36.6%) suffered from parasitism by Eulophidae. The increase in the survival rate and the decrease in the parasitism rate by Eulophidae occurred simultaneously in significant possibility (bootstrap possibility, *P* < 0.001). Furthermore, the egg predation rate was significantly lower in experimentally rolled leaves than in unrolled leaves (bootstrap possibility, *P* < 0.001). The increase in the survival rate and the decrease in the egg predation rate occurred simultaneously in significant possibility (bootstrap possibility, *P* < 0.001). However, in experimentally rolled leaves, mortality by herbivory was significantly higher than that in unrolled leaves (bootstrap possibility, *P* < 0.001). Herbivory was mainly caused by lepidopteran larvae estimated by infestation form or remaining frass in leaf rolls, as well as direct observation of larvae.

## Discussion

In this study, we showed that simple modification of leaves, that is, leaf rolling, caused marked changes in the fate of immature attelabid weevils related to natural enemies. In particular, a decrease in the parasitism rate by Eulophidae and the egg predation rate contributed to the increase in the survival rate. The fact that parasitism rate by Eulophidae in experimentally rolled leaves was 0% compared to that in unrolled leaves (36.6%) suggests that leaf roll acted as an “insuperable barrier” against Eulophidae. This result is consistent with our previous study revealing that leaf rolling species in Attelabidae were less parasitized by eulophid wasps^16^. The reason why eulophid wasps do not parasitize eggs or larvae in experimentally rolled leaves may be explained by two different hypotheses: failure-in-access and failure-in-finding. The failure-in-access hypothesis is that eulophid wasps can find hosts and attempt to parasitize, but the leaf roll acts as a structural barrier and eulophid wasps cannot reach weevil eggs or larvae. Considering that leaf rolls in this experiment were loosely rolled and oviposition sites would be easy to access, the plausibility of this hypothesis seems relatively low. On the other hand, the failure-in-finding hypothesis is that eulophid wasps cannot find hosts in leaf rolls because they cannot recognize a “rolled leaf” as a target structure containing hosts. Eulophidae are known as one of the dominant parasitoids of various leaf miners such as leaf mining moths, flies, sawflies, or beetles^20,21^. Some parasitoids of leaf miners have been reported to have evolved specific visual searching traits for leaf miners during flight^22–25^. For example, parasitoids were more attracted to the leaves with many leaf mines using visual cues^25^. In addition to visual cues, parasitoids of leaf miners also use chemical and vibrational cues for host searching, similar to other parasitoids^26–28^. Considering that in the present study, chemical and vibrational cues would not differ between experimentally rolled and unrolled treatments, changes in the visual cues likely affected the search success of eulophid wasps. In our study, of the 36.6% of eggs and larvae from unrolled leaves parasitized by Eulophidae, 25.6% was attributable to egg parasitism. This means that mine shape would not be an important visual cue in this case because no mine existed on leaves in the egg stage. Thus, eulophid wasps might have a host searching image of “cut-off leaf on the ground”, which was basically flat and thin, and leaf rolls were not recognized as a host because the shape differed from the searching image. The protective barrier effect of leaf roll for inner insects has been reported previously^1,5–15^. However, this study suggests that the leaf roll effect is not only a structural barrier but also a “visual modification” itself. In order to confirm this visually protective effect of leaf rolls, further experiments controlling leaf shapes in various patterns and comparing parasitism rates among treatments are needed. In this study, important information on the timing of parasitoid attack was also indicated: Mymarid wasps and *Ophioneurus* sp. were suggested to attack hosts on the tree shortly after weevil oviposit into the leaf and before the leaf was completely cut from the tree. The time from oviposition to leaf cutting is reported to be approximately 30 min in a rhynchitin non-leaf-roller species, *Deporaus septemtrionalis*^29^. It is surprising that parasitoids can finish their host finding and oviposit in such a brief time and probably represents a product of the arms race between parasitoids and weevils^16^. In addition, the weevil behavior of cutting leaves from the host tree might contribute to avoidance of heavier parasitism rates. If leaves including eggs remain suspended from the tree, the success rate of parasitism would most likely increase due to prolonged opportunity for parasitoids to attack.

The other factor related to the increase in survival rate of immature weevils was the decrease in mortality due to egg predation. Our results indicate the presence of predators on the ground that feed primarily on eggs in leaf tissue instead of on eggs in leaf tissue within leaf rolls. Few studies have revealed predators of leaf miners in the egg stage, and most studies of leaf miner mortality focus on larvae^30^. Digweed^31^ reported potential egg predators of a leaf mining sawfly as spiders, staphylinid beetles, coccinellid beetles, Hemiptera, and thrips. However, in this study, we should consider potential egg predators not on the tree but on the ground. From the soil meso-organisms and macro-organisms lists, predators and opportunistic predators in the litter would be mites, Opiliones, Isopoda, centipedes, millipedes, ground beetles, and spiders^32^. Furthermore, ants and dipteran larvae could also be considered as potential predators or opportunistic predators^33,34^. Eggs in unrolled and rolled leaves were protected in the leaf tissue, but leaf decomposition or egg dislodgement by soil organisms might occur more easily in unrolled leaves than in rolled leaves. Such decomposition or dislodgement causes exposure of eggs and a higher risk of predation. Thus, eggs in rolled leaves might show lower predation rates than those in unrolled leaves.

In contrast to predation, weevil mortality due to herbivory, especially by moth larvae, increased in experimentally rolled leaves compared to unrolled leaves. Thus, leaf rolls are not only protective refuges but also potentially risky hiding places for immature weevils. Our observations could be attributed to the fact that leaf rolls were preferred by herbivorous moth larvae as shelters; leaf shelters, that is, leaf rolls, leaf galls, leaf folds, or leaf ties, are often preferred and secondarily used by several insect species, sometimes providing them with a protective effect^13,35–38^. The reason why previous studies on the effect of leaf rolls did not detect the negative effect of herbivory could be that the observed leaf rolls were constructed by lepidopteran larvae that could escape herbivory and construct new leaf rolls. In the litter on the forest floor in Japan, lepidopteran larvae, such as those belonging to Blastobasidae or Tineidae, crawl while searching for fallen leaves to feed on. In an attelabid species, *Cycnotrachelus roelofsi* (Attelabinae), *Neoblastobasis spiniharpella* (Blastobasidae) larvae were found to infest the inside of leaf rolls; as a result, weevil larvae sometimes died of direct infestation or the lack of food^34^. In such cases, leaf roll construction had a negative effect on immature survival. However, in the species of Attelabinae, leaf roll construction is crucial to avoiding egg parasitoids, while mortality by herbivory is not so high^34^. Thus, the protective effect of leaf rolling against egg parasitoids exceeds the negative effects of herbivory. Further, leaf roll construction using excessive leaves by some Byctiscini species (Attelabidae, Rhynchitinae) may be a counter evolution to avoid mortality by herbivory. Various lepidopteran species and dipteran species emerge from leaf rolls of some Byctiscini species consuming leaf rolls (Kobayashi C, unpublished data), and competition for leaf roll resources sometimes causes larval death because weevil larvae cannot exit leaf rolls and search for new leaves. Thus, excessive leaves in the leaf roll may save weevil larvae from dying from food loss or infestation because of herbivory.

Regarding environmental stress, we did not detect any effect of leaf rolls. This may be because immature weevils in unrolled leaves were not directly exposed to environmental stresses due to their leaf mining habit. Therefore, both rolled and unrolled treatments experienced the same environmental conditions.

In summary, the survival rate of the attelabid weevil in this study was significantly increased by leaf rolling. Thus, this study suggests that selection pressure to evolve leaf rolling behavior still exists in the natural population, at least in Attelabidae. However, whether the leaf rolling behavior evolves will depend on the balance of positive and negative effects of leaf roll, that is, the degree of pressure exerted by parasitoids, predators and herbivores. Furthermore, constructing leaf rolls incurs energy costs and time for oviposition. Considering that most Deporaini species are less than 5 mm in length, the behavioral costs for rolling leaves would be high. In Deporaini, few species evolved leaf rolling traits independently, while the others were leaf miners in cut leaves and did not roll leaves^17^. Although the total survival rate was higher in rolled leaves than in unrolled leaves, contradictory effects of leaf rolls added to construction costs may explain this sporadic evolutionary pattern in leaf rolls of Deporaini. If leaf rolling traits have a mostly positive effect on egg and larval survival, leaf rolling behavior may have evolved more frequently or further diversification of leaf rolling species may have occurred.

## Materials and methods

### Study species

*Apoderites commodus* (Coleoptera; Attelabidae; Deporaini, Fig. 1a) is a “cut” leaf miner, i.e., female weevils oviposit into leaf blade tissue creating a slit (Fig. 1c, d), cut it off from the tree (Fig. 1b), and then hatched larvae grow inside the leaf tissue on the ground by feeding on inner leaf tissue without being exposed or moving to the outside of the infested leaf. Usually, one egg or two eggs are laid into the leaf tissue. After leaf mining for 1 or 2 months, mature larvae exit the leaf, pupate in the soil, and then emerge the next spring. Plant-cutting behavior for oviposition by female insects is a unique trait of Attelabidae acquired once by the common ancestor of Attelabidae^16^, and is considered an adaptation to avoid chemical defenses of fresh or intact plants towards immature insects.

### Study site and experiment

**Figure 4:**
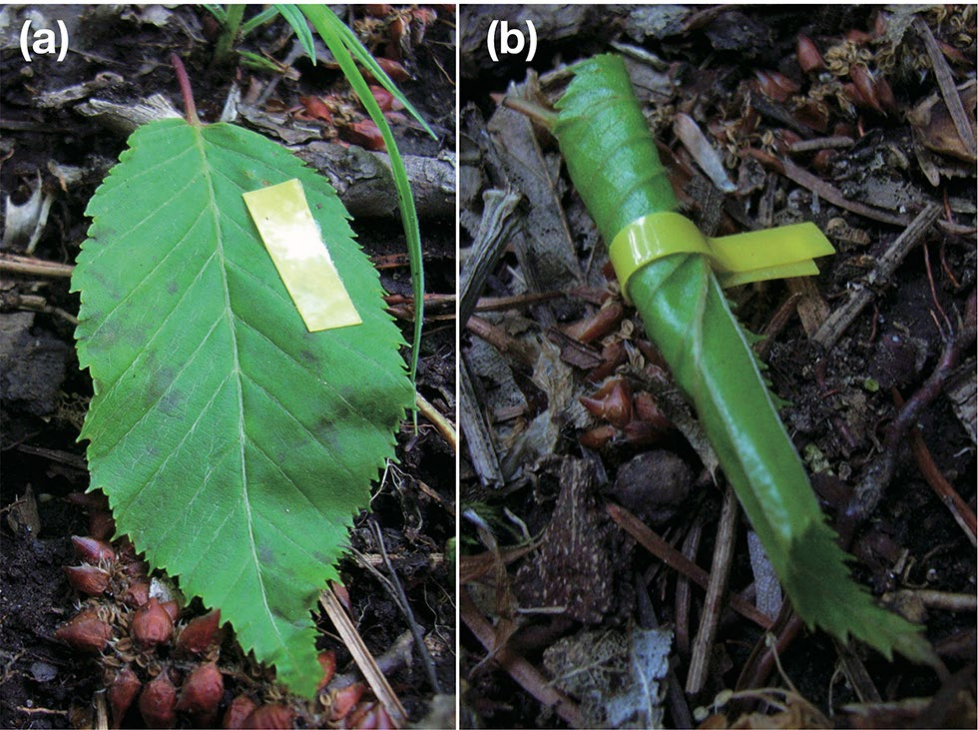
(**a**) A cut leaf where *A*. *commodus* had oviposited labeled with colored tape (unrolled treatment). (**b**) An experimentally rolled leaf found after a female *A*. *commodus* weevil oviposited and cut the leaf from the host tree (rolled treatment).

We collected 642 cut leaves of *Carpinus laxiflora* (Betulaceae) from the ground, in which *A*. *commodus* oviposited (Fig. 1b) at Yomogida (38.45° N, 140.46° E), Miyagi, Japan on 21, 23, 24 and 26 June 2011. Study site was temperate deciduous forest dominated by *Q. serrata* and *Castanea crenata* at 170 m elevation. Of the 642 collected leaves, 50 were taken to the laboratory in order to check survival or mortality rate before the experiment and were individually kept in an incubator at 20 °C. Of the other collected leaves, 319 leaves were only labeled by plastic tape with no leaf roll treatment (Fig. 4a), and 273 leaves were experimentally rolled using plastic tape (Fig. 4b), before being placed on the ground as similar to natural dispersal as possible. Experimental leaf rolls were loosely rolled and we took care not to cover the weevil’s oviposition holes with the leaf layer. Note that in this case, contrary to other common leaf rollers, weevil larvae are leaf miners and therefore would be “mining” in artificial leaf rolls. In addition, for leaves with no leaf roll treatment, eggs and larvae would not be exposed to external surroundings as internal leaf feeders. Thus, comparison between leaf-rollers and non-leaf rollers in this study indicate the effect of leaf rolls for internal feeders.

The placed leaves were recollected on July 14, 2011, approximately 3 weeks after placement, by which time the larvae were nearly ready to pupate but had not yet exited the leaves for pupation. Of the 273 experimentally rolled and 319 unrolled leaves with marking, 212 and 273 leaves were recollected, respectively. The recollected leaves were taken to the laboratory and the developmental stage (egg or larva), status (living or dead), and the cause of mortality were recorded for each individual. If a larva was still alive in the leaf, it was kept individually in an incubator at 20 °C until an larva or a parasitoid emerged. Emerged larva was kept individually in the soil for pupation in an incubator at 20 °C for three months, at gradual winter condition for four months (4 °C), and then at gradual spring condition (20 °C) until adult weevil or a parasitoid emerged. These recollected leaves contained 212 eggs in the experimentally rolled treatment and 270 eggs in the unrolled treatment.

### Mortality classification

Egg and larval mortalities were classified into seven categories: unknown larval death, unknown egg death, egg predation, herbivory, parasitism by Eulophidae, parasitism by Mymaridae, and parasitism by *Ophioneurus* sp. (Trichogrammatidae). Parasitoids were identified by specimens that emerged from weevil eggs or larvae using a stereomicroscope. We categorized the mortality as unknown egg death and unknown larval death when we found dead eggs or larvae without external damage; unknown egg death and unknown larval death were caused not by external factors such as predators or parasitoids, but by inner factors such as developmental failure, intrinsic damage, poisoning, and so on. Unknown egg death and unknown larval death would contain mortalities due to abiotic factors such as temperature, relative humidity, precipitation, etc. We categorized the mortality as predation when we could not find any egg or larva in leaves without herbivory. Herbivory is a mortality caused by leaf infestation mainly by moth larvae. In such leaves, weevil eggs or larvae would die because of direct infestation or a lack of food. In most cases, moth larvae had already exited from recollected leaves before the sampling. Thus, in such cases, herbivory by moth larvae were identified only by the frass or moth larval exuviae in leaves. Some moth larvae were found in recollected leaves and were kept in the laboratory, but moth species could not be identified because of failure in adult emergence. Missing samples were excluded from the statistical analyses because their status could not be ascertained and were considered not to interact with mortality differences between treatments. Survival and mortality rates between treatments were analyzed by non-parametric bootstrap with 10^6^ replicates using R ver.3.2.1 (https://www.R-project.org).
